# Quality of life of family caregivers of stroke patients: A systematic review of qualitative research

**DOI:** 10.12669/pjms.41.4.11136

**Published:** 2025-04

**Authors:** Hongyun Chen, Norafisyah Makhdzir, Wei Chao Loh, Lining Wang

**Affiliations:** 1 Hongyun Chen Department of Nursing, Faculty of Medicine and Health Sciences, Universiti Putra Malaysia, 43400, UPM, Serdang, Selangor, Malaysia; 2 Norafisyah Makhdzir Department of Nursing, Faculty of Medicine and Health Sciences, Universiti Putra Malaysia, 43400, UPM, Serdang, Selangor, Malaysia; 3 Wei Chao Loh Department of Medicine, Faculty of Medicine and Health Sciences, Universiti Putra Malaysia, 43400, UPM, Serdang, Selangor, Malaysia; 4 Lining, Wang Department of Nursing, Faculty of Medicine and Health Sciences, Universiti Putra Malaysia, 43400, UPM, Serdang, Selangor, Malaysia

**Keywords:** Caregivers, Evidence-based nursing, Qualitative research, Stroke, Systematic review

## Abstract

**Objectives:**

Stroke imposes a substantial burden on individuals and families. The caregiving role for stroke survivors is often assumed by family members. This systematic review aimed to synthesize qualitative studies that explored the lived experiences of family caregivers of stroke patients, thereby determining the breadth and depth of qualitative evidence on their experiences.

**Methods:**

The review included studies published between February 2009 and February 2024, following the PRISMA guidelines for Systematic Reviews. The following databases were searched: PubMed, Web of Science, Scopus, MEDLINE, ScienceDirect, HINARI databases for qualitative research exploring the experiences, needs, and burdens experienced by caregivers of stroke patients.

**Results:**

Eleven studies were systematically reviewed, revealing three primary themes: the multifaceted burdens experienced by family caregivers of stroke patients and the positive aspects of caregiving, including identified needs and sources of support.

**Conclusion:**

Healthcare professionals should prioritize the psychological well-being and caregiving needs of stroke survivors’ caregivers. Comprehensive support, including psychological counseling and professional training, should be provided to enhance their caregiving abilities. Additionally, governments and healthcare institutions should offer family-centered support to ensure the quality of life for both patients and their caregivers.

## INTRODUCTION

Stroke is an acute cerebrovascular disease caused by the rupture of cerebral blood vessels or blockage of blood flow to the brain, resulting in brain tissue damage. Characterized by high incidence, high disability rate, high recurrence rate, high mortality rate, and high disease burden, stroke poses a significant threat to the health of the Malaysian population.

Malaysia, an emerging economy in Southeast Asia with a population of approximately 32.6 million, with an annual population growth rate of approximately 0.6%, is facing an increasing public challenge due to stroke. According to the National Neurological Registry of Malaysia (NNEUR), the incidence of stroke cases in Malaysia is rising^1^ and stroke is the third most common cause of death and ranks among the highest in disability rates in the country.^2^

In 2019, data from Malaysia showed a rising trend among strokes with incidence 47, 911 (UI 43,757–52,839); deaths 19,928 (95% UI: 15,909–25,000); prevalence 443,995 (95% UI: 414,703–476,838); DALYS 512,726 (95% UI: 420,450–629,695).^3^ The number of patients with stroke-related disability increased. At the national level, the incidence of strokes increased by 4.9% among men and decreased by 3.8% among women between 2008 and 2016. The 28-day all-cause death rate decreased for both sexes by 13.1% and 10.6%, respectively.^4^

Caregivers play a crucial role in supporting stroke patients, who may suffer from various impairments in movement, language, and cognition and more.^5^ However, caregivers are often unprepared for their new role caring for their family member who has suffered from a stroke. This lack of preparation can lead to distress and negatively impact their physical, mental and social health.^6^

Caregivers provide essential communication and emotional support not only during the patient’s hospitalization but also throughout the daily care and rehabilitation process after discharge.^7^ The high costs of long-term medical care, uncertainty surrounding the patient’s prognosis, and the prolonged nature of caregiving responsibilities frequently exacerbate the psychological burden on caregivers, leading to anxiety and negative emotions.^8^ These factors can further affect the quality and effectiveness of the care provided. Although numerous qualitative studies have explored the lived experiences and needs of family caregivers of stroke patients both domestically and internationally, a single qualitative approach cannot fully capture the complex realities faced by this group. Given the cultural differences between Malaysia and Western countries, more in-depth research is needed to comprehensively explore the needs and experiences of this population.

To address this gap, this review analyzed a substantial body of existing research on caregiver resilience, particularly in the context of stroke patients. By synthesizing these findings and presenting comprehensive results, this review aimed to provide valuable insights to support caregivers in Malaysia. This study analyzed the caregiving experiences and underlying needs of stroke caregivers to identify gaps in clinical care and disease management. In doing so, it offers a more comprehensive theoretical and practical foundation for improving the support provided to caregivers across Malaysia.

## METHODS

A systematic review of qualitative studies on the caregiving experiences of caregivers of stroke patients was conducted. This review followed the PRISMA (Preferred Reporting Items for Systematic Reviews and Meta-Analyses) guidance and systematically searched for published qualitative research on the caregiving experiences of stroke caregivers. The study extensively explored caregivers’ lived experiences in caring for stroke patients and assessed their actual needs and burdens from the caregivers’ perspective.

The PubMed, Web of Science, Scopus, MEDLINE, ScienceDirect, and HINARI databases were used for bibliographic exploration. Studies released throughout the last 15 years, from February 2009 to February 2024, were considered in the review. Only English-language qualitative empirical studies were selected. The bibliographic search was conducted using the following English Medical Subject Headings (MeSH) terms: “Caregivers” (population), “Stroke” and “Cerebrovascular Disorders” (condition), and “Qualitative Research,” “Empirical Research,” and “Caregiving” (processes). The search strategy used was:”(Caregivers) AND (Stroke OR Cerebrovascular Disorders) AND (Qualitative Research)”

**Fig.1:**
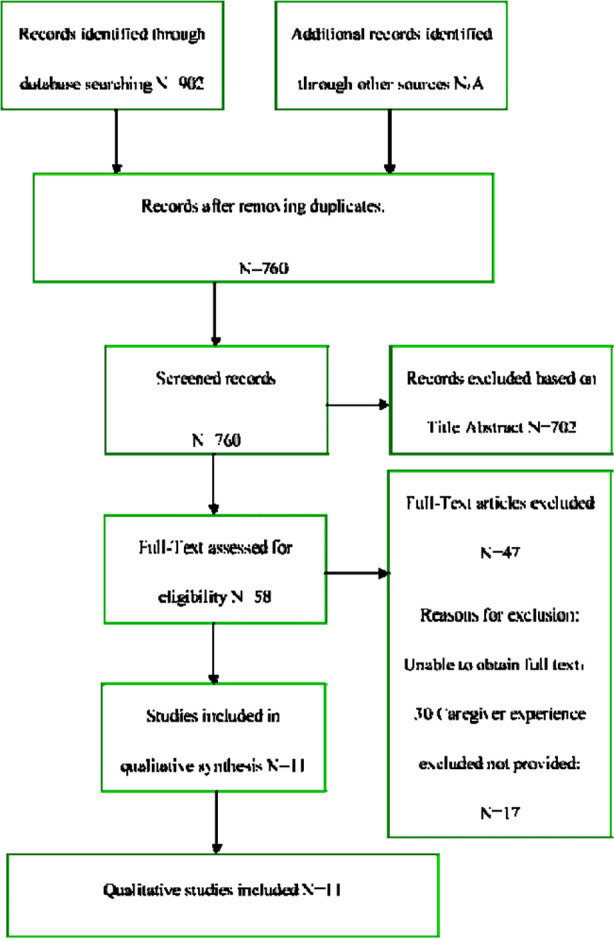
PRISMA diagram.

The flow diagram of the search process is presented in Fig.1. Initially, 902 articles were identified through bibliographic exploration in the following databases: PubMed (153), Web of Science (198), Scopus (176), MEDLINE (124), ScienceDirect (139), and HINARI (112). After applying the inclusion and exclusion criteria, 58 articles were pre-selected. In cases of disagreement, two of the four researchers attempted to reach a consensus on whether to include studies. Thirty studies were excluded because the full text was not available; another 17 studies were excluded because they did not explore the real experience and needs of caregivers (i.e., they failed to help caregivers adapt to and cope with the disease process). One researcher reviewed the full text of the pre-selected studies, and 11 articles were finally included^9^-^19^ (Fig.1 PRISMA diagram).

The quality of the articles was evaluated using the criteria proposed by Hawker et al. A review was conducted for each study, assessing the following areas: title and abstract, introduction and objectives, methodology, sample, data analysis, ethical considerations, results, generalizability and transferability, and practical implications. Each aspect was scored on a Likert scale from zero to four, with four indicating high quality and zero indicating very low quality. A total quality score was then calculated for each study (Table-II).

**Table-I:** Description of Articles Included in the Review

S. No.	Author (year)	Design	Participants	Phenomenon of interest	Interview location	Main results
1.	Kitzmüller et al.^11^	Phenomenological research, semi-structured interviews	Family caregivers of 22 stroke patients	Spouses and children’s experience of long-term family life and care experience with stroke patients	Respondent’s home	4 themes: The key role of family support; The emotional bond between parents and children; The challenges of marriage after stroke; Couples coping with change together
2.	Jones et al.^12^	Phenomenological research, semi-structured interviews	Family caregivers of young stroke patients	Care experience of family caregivers of stroke patients	Respondent’s home	4 themes: emotional experience of caregiving; importance of parental care; negotiation of independence and dependence; impact of stroke on family relationships
3.	*Yasmeen El Masry* et al.^13^	Phenomenological research, semi-structured interviews	Three in-depth face-to-face semi-structured interviews were undertaken with eleven dyads	Elderly stroke survivors and their spouses		Five themes were identified: caregiver attributes and coping strategies; limitations of stroke survivors; outside employment and financial stressors; and unexpected positive changes in relationships and priorities.
4.	Lawrence et al.^14^	Phenomenological research, semi-structured interviews	Family caregivers of stroke patients	Care experience of family caregivers of stroke patients	Respondent’s home	3 themes: psychological burden of caregivers; impact of stroke on family relationships; confusion and reorientation of caregivers
5.	Chen PC et al.^15^	Phenomenological research, semi-structured interviews	Family caregivers of stroke patients	Psychological experience and coping style of spouses of stroke patients during caregiving	Doctor’s Office	4 themes: multidimensional stress load; impact on life; different coping attitudes; urgent need for social support
6.	Yuan Z et al.^9^	Phenomenological research, semi-structured interviews	Family caregivers of stroke patients aged 25-50	Positive experience of primary caregivers of stroke patients in hospital-based humanistic services during their care of patients		3 themes: factors that contribute to the positive experience of hospital humanistic services; positive experiences brought about by hospital humanistic services; caregivers’ expectations of hospital humanistic services
7.	*Esther OW Chow* et al.^16^	Phenomenological research, semi-structured interviews	Twenty Australian informal caregivers and 10 stroke survivors	Elderly stroke survivors and their spouses	Community	Five themes: changes and difficulties in life after stroke; coping strategies and adaptation; impact on marital relationships; supportive internal resources in life, such as beliefs, values, and motivations; and social policies and services.
8.	Cameron et al.^17^	Phenomenological research, semi-structured interviews	Family caregivers of middle-aged stroke patients	Caregiving experience, experience, burden and coping strategies of family caregivers of stroke patients		3 themes: caregiving experience and experiences of middle-aged family caregivers; impact of stroke on life; loneliness and social alienation of caregivers
9.	Haji-Mukhti et al.^18^	Phenomenological research, semi-structured interviews	Family caregivers of middle-aged and elderly stroke patients	Caregiving experience, experience, burden and coping strategies of family caregivers of stroke patients		5 themes: Post-stroke care experience during the COVID-19 pandemic; Challenges faced by family caregivers; Positive coping strategies for caregivers; Emotional support; The power of faith
10.	Fen XY et al.^10^	Phenomenological research, semi-structured interviews	Family caregivers of young and middle-aged stroke patients	The dual coping experience of stroke patients and their spouses	Ward	4 themes: lack of knowledge at the onset of symptoms; changes and adaptations during the diagnosis and treatment of the disease; challenges faced in dealing with the disease in the second hospital; and active solutions sought in dealing with the disease in the second hospital
11.	Xuan G et al.^19^	Phenomenological research, semi-structured interviews	Family caregivers of middle-aged stroke patients	Sense of benefit from illness among family caregivers of stroke patients	Stroke Clinic	3 themes: Improving personal abilities; Cultivating new life beliefs; Integrating into life and actively sharing

**Table-II:** Quality Assessment of Articles Included in the Review.

S. No.	Author (year)	Abstract and title	Introduction and Aims	Method and Data	Sampling	Data Analysis	Ethics and Bias	Results	Transferability /Generalizability	Implications and Usefulness	Total Score
1.	Kitzmüller et al.^11^	4	4	3	4	4	4	4	3	4	34
2.	Jones et al.^12^	4	4	4	4	3	3	4	4	3	33
3.	*Yasmeen El Masry* et al.^13^	4	4	3	4	4	4	4	4	4	35
4.	Lawrence et al.^14^	3	4	3	4	3	4	3	4	3	31
5.	Chen PC et al.^15^	4	4	4	4	4	3	3	2	3	31
6.	Yuan Z et al.^9^	3	4	3	4	4	3	3	2	3	29
7.	*Esther O W Chow* et al.^16^	4	3	3	3	2	4	2	4	2	26
8.	Cameron et al.^17^	3	4	4	3	4	3	3	4	3	31
9.	Haji-Mukhti et al.^18^	4	4	4	4	4	4	4	4	3	35
10.	Fen XY et al.^10^	4	4	4	3	4	4	4	3	3	33
11.	Xuan G et al.^19^	4	3	3	4	3	3	3	3	2	28

a Hawker’s criteria for quality assessment. Maximum score ¼ 36.

The data search was conducted by C. and W., who systematically identified relevant qualitative studies. To ensure accuracy and reliability, the extracted data were independently cross-checked by Chen and Wang. Additionally, full-text reviews were performed by N. and L. to confirm the inclusion criteria and assess the quality of the selected studies. Any discrepancies were resolved through discussion among all authors.

### Research design of the studies review:

All the literature included in this article adopted the phenomenological research method and studied the caregivers of stroke patients through semi-structured interviews (Table-I).

### Multiple burdens faced by family caregivers:

### Psychological burden:

Family caregivers bear a significant psychological burden when caring for stroke patients, one caregiver expressed, “I just want to get out of the house and be alone, I’m tired of all the responsibilities”^11^; another shared, “I hired a caregiver to take care of him during his hospital stay, but the psychological burden was too great. I felt exhausted and often felt overwhelmed.”^15^ Addition to the physical and emotional demands, caregivers often experience anxiety and depression related to their loved one’s health conditions, one caregiver explained: “The worst part is that I can’t communicate and discuss things with him. It’s very disturbing. No one can imagine how helpless I feel.”^14^ another said: “It’s very difficult. Since the onset of the disease, there hasn’t been a day that I haven’t worried about whether it will happen again today.”^11^ These experiences further increase the psychological burden on caregivers, causing them to fall into a state of emotional fatigue and self-sacrifice. These emotional challenges only intensify the psychological strain, pushing caregivers toward emotional fatigue and self-sacrifice. The burden is not solely due to concerns about the patient’s condition but is also worsened when caregivers’ own needs are neglected, as one caregiver noted, “After work, all I want is to rest, but when I get home, I still have to take care of him, and it feels exhausting.”^15^

### Physical burden:

Caring for stroke patients often involves significant physical demands, including helping with basic activities of daily living, such as bathing, dressing, and eating. This creates a substantial burden for caregivers, one caregiver shared, “I have to go home to bathe my father, and I don’t have enough time to rest.^18^” Another said, “I have to take my grandson to piano lessons, go home to cook for my wife, and I still need time to rest.” These responsibilities frequently leave caregivers physically exhausted. Furthermore, the physical burden may be compounded by the patient’s limited mobility, especially when rehabilitation and movement support are required, as one caregiver described, “His recent rehabilitation has been so intense that I’ve had little time to rest. I’ve also developed high blood sugar and high blood pressure.”^10^

### Financial burden:

Financial burden is another serious problem that family caregivers of stroke patients generally face. Given the need for long-term care and rehabilitation, caregivers often have to invest significant time and financial resources, one caregiver explained, “During the epidemic prevention and control period, our financial situation was affected, and life became extremely difficult. My children needed to pay tuition, so the funds for my husband and I to stay in the hospital had to be reduced”^18^. Lots of caregivers need to give up their jobs or reduce their working hours to take care of the patient full-time, as one caregiver said, “In order to take care of him, I had to give up my job and lost my source of income. The doctor recommended that I stop eating first, but the specific recovery schedule has not been determined”^15^, these circumstances further add to the financial difficulties that the family may face.

### Caregiver Necessities and Support:

### Demand for Professional Knowledge:

Caregivers often have a limited understanding of medical terminology and professional knowledge, which results in insufficient or missing information about disease progression and prognosis, as one caregiver noted, “I heard some terms that the doctor used, but I didn’t really understand them.^17^” This lack of understanding has led to critical misjudgments regarding the patient’s condition. For instance, one caregiver recounted, “I woke up to find that he was in poor condition, and at that moment, I mistakenly thought he was just asleep, which nearly caused a delay in recognizing his illness.^10^” At the same time, medical staff failed to provide adequate guidance and information support, one caregiver noted that “Most of the information I got from medical staff was what I happened to hear during their shifts^15^”; another caregiver mentioned that “Health care professionals are limited to completing their work and then leaving in a hurry^10^,” causing caregivers to feel confused in the care process. All caregivers agreed that health care professionals should be more proactive in providing disease-related expertise to family members, one caregiver suggested “It would be really helpful if there was an information booklet or online resource that listed the various possible effects of stroke, because right now, other than her limited movement, I don’t know what else I can do for her”.^17^

### Demand for Social Support:

Social support plays a crucial role in the caregiving experience. Faced with immense psychological stress and emotional burden, caregivers eagerly look for understanding and support from society. This support is not limited to the comprehensive rehabilitation facilities, programs, and processes provided by professional institutions. As one caregiver remarked, “It didn’t seem to help much; there was just some initial consultation, and then a week later, the patient was notified to be discharged”.^14^ “I hope the rehabilitation process is arranged more closely to better accommodate the daily activities my daughter needs to undertake”.^9^ Moreover, social support also encompasses the economic burden of medical and rehabilitation costs for patients. One caregiver stated, “My daughter just had a baby, my son is 30 and still hasn’t married, and we also need to buy a house. Every day, I check the expense list, and there are some large expenses that are not reimbursed”.^10^ Another added, “I hope the health insurance policies can pay more attention to people like us”.^10^

### Demand for Emotional Support:

During the caregiving process, caregivers often confront the severity of the patient’s illness and the demanding nature of their caregiving tasks, which leads to psychological burdens and feelings of isolation. To alleviate these mental strains, caregivers urgently need support and companionship from family and friends. One caregiver stated, “I have a large family, which means a lot to me. Family will always support you; you have to keep going”.^11^ Another shared, “We hope to have a nearby place because I want my sisters to be close by”.^17^ In addition, caregivers emphasize the urgent need for attention and intervention regarding the patient’s psychological state, noting that healthcare professionals play a crucial role in reassuring caregivers.

One caregiver expressed, “I’m really worried about my son’s mental state: he’s having a hard time coping with this situation. If every doctor and nurse could do a little more psychological support during treatment, I think it would be very helpful”.^9^ While focusing on the patients, caregivers also emphasize the importance of addressing the psychological stress faced by family members and providing them with emotional support. One caregiver noted, “We feel very sad, and without the support of healthcare staff, our needs go unmet”.^11^ Another stated, “I call for increased attention to the mental health of family members, along with psychological counseling for them, because we also bear significant psychological pressure”.^9^

### Positive psychological experience of caregivers:

In the process of fulfilling their caregiving responsibilities, caregivers exhibit a variety of positive coping strategies, primarily reflected in their expectations and support for the patient’s recovery. Specifically, caregivers emphasize the importance of the patient’s active participation in rehabilitation, focusing on improving their current situation rather than passively accepting it. One caregiver stated, “I hope he makes more efforts to improve, rather than just accepting his current condition”.^12^ This optimistic outlook helps patients better face the various challenges during the rehabilitation process. During rehabilitation, caregivers provide emotional support and take proactive measures to help patients maintain a positive attitude. As one caregiver noted, “When he realized what he couldn’t do, he became very frustrated. We continually encourage him to face things positively”.^17^ Despite facing immense physical and mental pressures, caregivers recognize the importance of their own well-being. They adopt a positive and optimistic approach to life, effectively managing various challenges through flexible strategies and a hopeful mindset. As one caregiver expressed, “She is relatively lucky; I now believe that happiness is most important and that we should go with the flow”.^15^ Another remarked, “There are always more methods than difficulties… This is also a form of companionship”^19^, while yet another stated, “I used to rely on him a lot, but with such sudden changes, I cannot afford to break down”.^10^ This positive attitude towards coping with stress injects a constructive momentum into the entire caregiving process.

## DISCUSSION

### The Mental and Physical Health of Caregivers for Stroke Patients:

The research results indicate that family caregivers of stroke patients generally face physical and mental health issues while fulfilling their caregiving responsibilities over the long term.^10^,^13^,^18^ According to the National Neurological Registry of Malaysia (NNEUR), approximately 56% of caregivers among caring the neurological patients experience anxiety symptoms, while 28% exhibit depressive symptoms.^1^ Moreover, the disease burden of stroke patients imposes significant financial and social challenges on their families, which in turn has a profound impact on the psychological well-being and social adjustment of stroke survivors. Caregivers not only endure physical, psychological, and economic pressures but also need to pay attention to whether the patients can successfully reintegrate into society. The elevated recurrence rate of stroke, coupled with the potential for physical disabilities^20^ to hinder social participation and exacerbated by increased financial strain on families, frequently results in caregiver burnout, anxiety, and feelings of helplessness.

This finding aligns with the research results of Li et al.^21^, where these negative psychological experiences directly affect caregivers’ physical and mental health as well as the quality of care they provide.^22^ Sutter-Leve’s research also pointed out that the state of physical and mental health is a key factor influencing caregiving ability.^23^ To effectively support family caregivers of stroke patients, it is imperative to prioritize their physical and mental well-being. This includes implementing strategies to alleviate their psychological burdens, recognizing their reasonable needs, and enhancing the quality of humanistic services in hospitals. Fostering open communication and cooperation among family members is also crucial. Furthermore, various sectors of society should actively engage in understanding caregivers’ unique psychological experiences and providing essential psychological counseling and emotional support.

### Importance of Support Strategies:

The research results indicate that family caregivers of stroke patients urgently need to strengthen support strategies to meet their multifaceted needs.^24^ This finding aligns with the research conducted by Wang et al.^25^, highlighting caregivers’ high demand for emotional adjustment, disease knowledge, rehabilitation guidance, and utilization of social resources. Family caregiving is crucial for the functional recovery and psychological well-being of stroke patients.^26^ Additionally, the integrated results reveal that most caregivers lack knowledge related to the disease and rehabilitation exercises, indicating an urgent need to enhance caregiving abilities, which is consistent with the findings of Leszko et al.^27^ Therefore, healthcare service institutions need to strengthen knowledge and skills training for caregivers by regularly organizing workshops and health education seminars to improve caregiving efficacy and confidence, thus promoting patient recovery.

Furthermore, many caregivers tend to reduce social activities as they shift their focus to the patient, leading to psychological health issues.^28^ Communities or institutions should provide professional rehabilitation services and establish WhatsApp or Telegram groups for family caregivers to alleviate psychological pressure. Good social support can offer caregivers material resources and emotional comfort, enhancing their ability to cope with negative events.^29^ Therefore, strengthening the support system requires the integration of resources from society, families, healthcare institutions, and the government to assist stroke patients and their caregivers in addressing the challenges of caregiving. The government should improve community rehabilitation facilities to meet the treatment and recovery needs of stroke patients, using financial funding and support from non-profit organizations to enhance the quality of caregiving services in healthcare institutions and reduce caregivers’ burdens.

### Positive Psychological Experiences and Role Adjustment:

The research results indicate that, during the caregiving process for stroke patients, both patients and their families perceive significant changes in their lives and successfully adjust their roles and effectively cope with stress to adapt to the new normal brought about by the illness. This finding is consistent with the research of Gallagher et al.^22^, which emphasizes the potential positive effects of traumatic events on individuals.^30^ For young family caregivers, relatively shorter life experiences provide them with more opportunities for personal development.^31^ When faced with the pressures brought on by illness, their inner resources are activated, leading to positive transformations in their worldview and values, and even experiences of post-traumatic growth.^32^ This positive cognitive and emotional response helps shape an optimistic coping mindset.^33^ By actively identifying and utilizing the positive experiences gained during the illness, caregivers can effectively enhance their emotional management skills and improve their ability to monitor and manage the disease.^34^

Throughout the course of the illness, continuous learning about stroke prevention and rehabilitation can help accumulate relevant information, thereby strengthening their ability to cope with and manage the disease. Moreover, this also aids in guiding caregivers to change their existing health beliefs, recognize the importance of maintaining a healthy lifestyle, and appreciate the support from family members and social resources, thereby reinforcing their self-efficacy when facing the challenges posed by the illness.^35^ Therefore, healthcare professionals should place particular emphasis on cultivating and promoting the positive psychology of family caregivers, enabling them to view their caregiving responsibilities with a positive attitude.

### Limitations

This study’s scope is limited to English literature, which may restrict the findings. Future research could consider incorporating studies from other languages to broaden the understanding of caregiver experiences. Furthermore, the lack of quantitative analysis in this study limits the ability to clearly delineate the specific impacts and regulatory roles of different factors on caregiver burden.

### Future Directions:

Future research should investigate the relationship between the severity of stroke in patients, social support, resilience, and caregiver burden. Additionally, longitudinal studies can offer deeper insights into the long-term effects of caregiver health on stroke outcomes. Finally, intervention studies can further analyze how various influencing factors affect caregiver burden.

## CONCLUSION

This Review examines the experiences, needs, and role adjustments of family caregivers for stroke patients as they fulfill their caregiving responsibilities. The aim was to raise awareness among healthcare professionals and society regarding this group, ensuring they receive the necessary support to help both patients and caregivers overcome various challenges. By improving the caregiving capacity of family members, the study seeks to enhance patient care and promote active recovery processes.
